# An Investigation of Cigarettes Smoking Behavior and Nicotine Dependence among Chinese Methamphetamine Users in Two Provinces

**DOI:** 10.1155/2014/175205

**Published:** 2014-06-15

**Authors:** Ziyun Wang, Yanping Bao, Shiyan Yan, Zhi Lian, Zhenjun Jia, Zhimin Liu

**Affiliations:** ^1^School of Public Health, Peking University, No. 38 Xueyuan Road, Haidian District, Beijing 100191, China; ^2^National Institute on Drug Dependence, Peking University, No. 38 Xueyuan Road, Haidian District, Beijing 100191, China; ^3^Institute of Basic Research in Clinical Medicine, China Academy of Chinese Medical Sciences, 16 Nanxiao Jie, Dong zhi men nei, Beijing 100700, China; ^4^Department of Criminal Science and Technology, People's Public Security University of China, Beijing 100038, China

## Abstract

*Objective*. To survey cigarette behaviors and nicotine dependence among Chinese MA users, explore risk factors for high nicotine dependence, and analyze the relationship between nicotine dependence and MA-related euphoria and sexual impulse. *Methods*. A cross-sectional study, applying a self-designed questionnaire with the Fagerström Test for Nicotine Dependence (FTND) and Visual Analog Scale (VAS), was performed among 391 MA users in Beijing and Guangdong, China. *Results*. Most MA users were smokers, including 159 having high dependence on nicotine (HD users, FTND > 5) and 197 low or medium dependent (LMD users, FTND ≤ 5). Men or married users were more likely to be highly dependent than women or unmarried users. Higher MA dose and ever-use of ketamine or alcohol were associated with higher likelihood of high nicotine dependence. HD users reported significantly higher euphoria and stronger sexual impulse after using MA, indicated by higher VAS scores. *Conclusions*. Potential risk factors for high nicotine dependence among MA users may include male gender, being married, higher MA dosage, and ever-use of ketamine or alcohol, which should be taken into consideration in individualized health promotion on smoking cessation. Severe nicotine dependence was associated with stronger MA-related euphoria and sexual impulse and it should be confirmed by further studies.

## 1. Introduction

Methamphetamine (MA), with street names of “glass,” “ice,” and “meth,” is one of amphetamine-type stimulants (ATS) with significant abuse potential and neurotoxic effects and it causes the release of central and peripheral monoamines, resulting in both physical and psychological alterations [1, 2]. MA can result in ATS-related disorders, including use disorder (characteristic of craving, recurrent stimulant use, tolerance, withdrawal, impact on social, occupational, or recreational activities, etc.), intoxication (e.g., significant problematic behavioral or psychological changes, tachycardia or bradycardia, pupillary dilation, elevated or lowered blood pressure, perspiration, or chills), and withdrawal (e.g., dysphoric mood and two or more of physiological changes like fatigue, vivid, unpleasant dreams, insomnia, or hypersomnia) [3].

According to World Drug Report 2013 published by United Nations Office on Drugs and Crime, ATS (excluding “ecstasy”) use remains widespread globally, with an estimated 33.8 million users (0.7 per cent of the global population aged 15–64) in 2011 [4]. In China, synthetic drugs (mainly ATS) users accounted for 38 percent of all registered users in 2012, higher than that in 2010 (28%) [4]. MA is the most commonly abused ATS, and data about seizures of ATS is mainly composed of MA in North America and East and South-East Asia [1, 4]. In 2010, MA seizures were more than double the amount in 2008, partly resulting from seizures increase in Central America and East and South-East Asia [5].

Cigarettes are commonly coused by MA users, and the prevalence of smoking in this population even exceeds 90% in some studies [6, 7]. Several studies show that nicotine may play an important role in MA use and the health effects on MA users [8, 9]. Smoking may be one important stage or inducing factor for using illicit drugs and early exposure to nicotine may precipitate the development of stimulant addiction [8–11]. Severe nicotine dependence may increase the risk of ATS users for depression with OR = 4.02 [12]. One animal study showed that nicotine may be acting through a nonassociative mechanism (at least in part) to reinstate MA-seeking behavior in rats [13].

As nicotine and MA have similar neurobiological basis in drug dependence-dopamine mediated rewarding system [14], MA and nicotine may have some interaction suggested by some studies [15, 16]. One animal study showed that the expression of immediate early genes (IEGs) in dopaminergic projection areas caused by nicotine and MA, when given in combination, was different from those elicited by each drug alone [15]. Another animal study found that nicotine and MA shared discriminative stimulus effects in some subjects and produced their interaction indirectly [16]. Moreover, previous studies have shown a correlation between psychostimulant-induced increase of extracellular dopamine in the striatum and self-reported measures of liking and “high” (euphoria) [17]; therefore, we hypothesized that the interaction between nicotine and MA might be reflected in the association between nicotine dependence and MA-related subjective effects (e.g., euphoria and sexual impulse), which play important roles in MA dependence.

Comprehensive knowledge of nicotine dependence and its relationship with MA-related subjective effects among MA users would be helpful for researchers and medical personnel deepen the understanding of the characteristics of MA, especially those tobacco cousers. However, there is no sufficient data to assist us in getting fully acquainted with the characteristics of nicotine dependence among MA users, especially in China. The purpose of this paper was (1) to investigate the cigarette behaviors and nicotine dependence among Chinese MA users and (2) to analyze risk factors in social-demographic characteristic and MA use pattern associated with high nicotine dependence and explore the relationship between nicotine dependence and MA-related subjective effects (euphoria and sexual impulse).

## 2. Materials and Methods

### 2.1. Sample

A cross-sectional study among ATS users was performed in compulsory and voluntary drug detoxification and rehabilitation centers in Beijing and Guangdong provinces, China, in 2010. Beijing lies in the north, while Guangdong province is located in the south. Both Beijing and Guangdong are economically developed and have large proportions of floating population [18, 19]. Estimation of the size of synthetic drug (MA, MDMA, and ketamine) users by Delphi methods was 30,000 (Beijing) in 2005 and 420,000 (Guangdong) in 2006 [20].

Participants should satisfy the following inclusion criteria [12]: (1) over 18 years old, (2) urine test positive for ATS drugs, (3) the criteria for ATS abuse or dependence in Diagnostic and Statistical Manual of Mental Disorders (DSM-IV; American Psychiatric Association), and (4) consented to participate. Participants, with serious physical illnesses such as severe cardiovascular disease, were excluded. Finally, a total of 391 patients who mainly abused MA were enrolled, among whom 356 were cigarette smokers (91.0%) and selected in the statistical analysis.

### 2.2. Measures

A self-designed questionnaire was applied in the study, and information (including social-demographic characteristics, pattern of drug use, attitudes and motivators towards ATS, subjective effects, impact on physiological function and behaviors after using MA, and smoking and drinking behaviors) was obtained from face-to-face interviews. In this paper, ever-use denotes the participant had ever used the substance before our survey started, but not necessarily concurrent with MA. Visual Analog Scale (VAS) was employed to depict subjective effects (euphoria and sexual impulse), with zero standing for no euphoria or sexual impulse and ten for the strongest feelings. Participants were asked to draw a fork in the straight line to represent their euphoria and sexual impulse after using MA.

The Fagerström Test for Nicotine Dependence (FTND) is a revision of the 8-item scale Fagerström Tolerance Questionnaire (FTQ, published in 1978) by removing item two (nicotine content of cigarettes) and item three (Do you inhale?), because they were found to be unrelated to biochemical measures of smoking dependence [21–24]. FTND has been widely utilized in public health researches to evaluate nicotine dependence. In FTND, the first and the fourth items are scored in a four-point system (0–3 points), while the rest four items scored in two-point system (0-1) [21]. As shown in Table 1, nicotine dependence was divided into low or medium nicotine dependence (0–5 scores, LMD) and high nicotine dependence (6–10 scores, HD) in our study, according to the total scores of FTND.

### 2.3. Statistical Analysis

Data entry was conducted twice with Epi Data software, version 3.1. Statistical analysis was performed with IBM SPSS Statistics software, version 20. Categorical variables (e.g., gender or education) were reported as percentage (%), while numerical data, including age and age at onset use, were presented as mean ± SD. Euphoria and sexual impulse were indicated in the form of median of VAS scores. Pearson chi-square test was utilized to analyze the association between demographic characteristics or drug use pattern and nicotine dependence, and Fisher's exact test was done if necessary [25]. Two-independent samples *t* test and nonparametric test model (Mann-Whitney *U* test) were employed to assess the ages and subjective effects (euphoria and sexual impulse), respectively. The binary logistic model was applied in the assessment of the association between nicotine dependence and potential risk factors identified significant in univariate analysis. Statistically significant findings were judged with a 2-tailed *P* value of 0.05.

### 2.4. Ethics Statement

This research protocol was approved by the Institutional Review Board of Peking University Health Center. Trained interviewers clearly explained the significance, aims, and content of the survey to the potential participants at the beginning of each survey and guaranteed that participants' private information would be kept secret. The face-to-face interviews were then conducted if the participants signed informed consent.

## 3. Results

### 3.1. Nicotine Dependence and Smoking Behaviors among MA Users

As shown in Table 1, of the 356 smokers, 197 (55.4%) were low or medium dependent on nicotine, and 159 were high nicotine dependent. Seventy-five percent of smokers often smoked the first cigarette within half an hour after waking up, and 66.3% smoked 1–20 cigarettes per day. 154 (43.3%) smokers found it difficult to refrain from smoking, and 207 (58.1%) still smoked even when they were ill in bed, while 188 (52.8%) thought they were reluctant to give up the first cigarette in the morning.

### 3.2. Social-Demographic Characteristics and Nicotine Dependence among MA Users

Most participants were male (76.1%) and Han people (88.6%) in our study (Table 2). 227 subjects (64.9%) had an education level lower than senior high school and approximately 10 percent finished their education in college or university. 235 subjects were unmarried (single or ever married but divorced or separated) when enrolled in our investigation. 145 smokers were unemployed (40.7%) and 112 were engaged in their private business (31.5%).

No significant difference was observed in education, ethnicity, occupation, age, and age of initial use of MA between LMD and HD users. HD users were elderly and tended to begin MA use later (with an average age of 28.9) than low-dependent ones (LMD), but not statistically different. However, there were significant disparities in gender and marriage status between LMD and HD users. Men (49.4%) were more likely to be highly dependent on nicotine than women users (28.9%), with a *P* value of 0.001. Lower proportions of HD users (32.3%) were observed in those unmarried (single and divorced or separated).

### 3.3. Association of Drug Use and Nicotine Dependence among MA Users

As shown in Table 3, 128 users had been in detoxification treatment before, occupying 36 percent of the whole subjects. Most MA users chose smoking as their main route of drug administration. The median MA dose of the whole subjects was 0.2 gram, and 166 users (47.3%) usually abused more than 0.2 gram per time. In our study, 20.7% of MA users ever used heroin and 19.2% ever used MDMA, 21.4% ketamine, and 53.3% alcohol.

Before this entry into treatment, participants had used MA for 8 months in median and the difference of cumulative use time between LMD and HD users was not significant (*P* = 0.522). Furthermore, in contrast with greater-MA-dose smokers, those in the smaller dose group (≤0.2 g) had less likelihood of becoming highly dependent on nicotine (36.8% versus 54.2%), with a *P* value of 0.001. Significant difference was observed between LMD and HD users in terms of other psychoactive substances. By comparison with that of those not using ketamine, the proportion of HD users was higher in those using ketamine (55.3 versus 41.9%). Higher percentage of HD users (59.4% versus 40.9%, 60.3% versus 41.1%, resp.) was also observed among those ever using heroin and MDMA. 53.3% of subjects had experiences of drinking, 50.5% of whom were HD users.

### 3.4. Multivariate Analysis of Risk Factors for Severe Nicotine Dependence

Significant factors identified by univariate analysis as shown in Tables 2 and 3 were included in a binary logistic regression model for nicotine dependence, but ever use of MDMA (“Ecstasy”) and heroin was excluded from the final version of equation as their* P* values were greater than 0.05.

The logistic regression analysis (Table 4) showed that gender (OR = 2.615) and marriage status (OR = 1.938) were associated with nicotine dependence. Those who usually used MA more than 0.2 g per time and those ever using ketamine or alcohol besides MA had higher likelihood of becoming highly dependent on nicotine, with ORs of 1.659, 2.044, and 1.699, respectively.

### 3.5. Nicotine Dependence and Subjective Effects (Euphoria and Sexual Impulse)

In Table 5, univariate analysis showed that, compared with LMD users, HD MA users reported significantly stronger euphoria after usual MA use or use for the first time, with higher VAS scores (5.20 versus 3.65 and 6.70 versus 3.00). Similarly, HD users reported stronger sexual impulse after using MA (7.60 versus 5.90).

In subgroup analysis with gender, HD users still reported significantly higher euphoria after onset and usual use of MA than LMD users, no matter male or female, with all *P* values smaller than 0.01. However, female HD users reported similar sexual impulse than female LMD ones (*P* > 0.05). Taking dose of MA into consideration, there still was difference between HD and LMD users in terms of euphoria of onset use and sexual impulse. But no significant difference was observed in self-reported euphoria after using MA usually among those using MA more than 0.2 g, *P* = 0.050.

## 4. Discussion

In our study, the prevalence of smoking among MA users was 91%, which was similar to previous studies among heroin users (99.4%) [26] and ATS users in Taiwan (91.5%) [6] and Zhejiang province (98.9%) [7], but much higher than that of general Chinese adult population (23%, estimated by WHO) in 2011 [27]. We did not retrieve any literature done to explore risk factors of nicotine dependence for MA users in China and our results illustrate that MA users who are male, married, using MA more than 0.2 g, and ever using ketamine and alcohol may be at higher risks of severe dependence on nicotine.

### 4.1. Nicotine Dependence and Smoking Behaviors among MA Users

Bao et al. reported that 67.2% of Chinese opiate addicts were highly dependent on nicotine (FTND score ≥7.0), with a similar range of age (30.4 ± 6.0) and a similar gender ratio (80.5% of male) to MA users in our research [26]. In our study, 55.4% of MA users had a FTND score of 5 or less and only 22.8% over 7, suggesting a lower dependence of MA users on nicotine compared with heroin users. In terms of cigarettes quantity, heroin addicts smoked 34.9 cigarettes per day on average during the phase of addiction [26], but only 18.0% of MA users smoked 31 or more cigarettes per day in our study.

Compared with studies among general population [28], the mean FTND score and proportion of severe nicotine dependence (FTND score >5) were much higher in our study. In a study of 1477 male smokers and 115 female smokers among Chinese dwellers [28], the average FTND score was 2.89 and only 27.1% were considered dependent (FTND ≥4). In our study, the percentages of participants smoked the first cigarette within 30 minutes after they woke up (75%) and participants reluctant to give up the first cigarette in the morning (52.8%) were also higher than those of the general population, implying a severe nicotine withdrawal among MA users [22, 28].

Moreover, MA dose was also found to be an independent risk factor for severe nicotine dependence in our study and those using MA more than 0.2 g/time had higher possibility to be HD users. In contrast, a previous study regarding heroin addicts found heroin dosage to be not significantly relevant with nicotine dependence, with OR = 1.02 (95% CI: 0.62–1.58) [26].

Our study suggests that MA may increase the cigarettes smoking and deepen nicotine dependence, which is consistent with some previous studies. One study [29] showed that* d*-amphetamine dose-dependently decreased the average duration of intervals between successive cigarettes smoked and increased the overall rate of smoking and preference for cigarette smoking over monetary reinforcement as well. Both 10 and 20 mg doses of amphetamine increased the number of cigarettes (1.4 ± 0.7, 1.8 ± 0.8, resp.) than during the placebo session [30]. Another study [31] revealed that methylphenidate (Ritalin, another kind of ATS) increased the total number of cigarettes smoked, number of puffs, and carbon monoxide levels as an orderly function of dose. In addition, among users of another stimulant-cocaine, cigarette smokers responded most strongly that using cocaine increased both the urge to smoke (78.8 ± 2.3, with 100 the strongest) and cigarette quantity (81.0 ± 2.3, with 100 the strongest) [32].

### 4.2. Analysis of Factors for Severe Nicotine Dependence

Some researchers [33–35] have found that males are more likely to be hardcore smokers (daily smokers with high nicotine dependence) than women. Consistent with those studies in general population, our study also showed that males have greater likelihood to be highly dependent on nicotine than females. Gender difference was also observed in tobacco smokers who used that and men had higher FTND scores and smoked more cigarettes [36].

In the study on heroin population [26], the number of times of relapse to heroin was positively related to nicotine dependence and those having relapsed to heroin 3 or more times had higher possibility to report severe nicotine dependence (OR = 1.89). However, in our study precise information of the relapse was not included in the questionnaire and only information on treatment times to detoxification center was collected. Our results displayed that 47.7% of MA users who had been treated more than once in detoxification center were highly dependent on nicotine, but this proportion was not significantly greater than that of those treating for the first time (43.0%).

Polydrug use was reported to be an independent risk factor (OR = 3.66, 95% CI = 2.08–6.45) associated with severe nicotine dependence among Chinese opiate addicts [26]. In our study, ever use of ketamine was proved to be associated with high nicotine dependence, with OR = 2.044. In one animal experiment, application of psychotomimetic ketamine concentrations to striatal slices augmented nicotine-evoked [^3^H] overflow and hyperactivity was observed after receiving ketamine injections for 30 days, which indicates that function of nicotinic acetylcholine receptors that mediate dopamine release is altered by ketamine [37]. More studies are still necessary to investigate the influence of ketamine on nicotine dependence.

Higher risks for severe nicotine dependence existed in those ever-use of alcohol, with OR = 1.699. A similar finding was reported in a case-control study, which found that 83 percent of alcoholics were smokers but only 34% among the nonalcoholic subjects [38]. In a cross-sectional epidemiological study [39] among Singapore residents, a positive association of alcohol abuse with nicotine dependence was significant with OR = 3.1. Another study revealed that alcohol significantly increased tobacco craving and tended to decrease the latency to start smoking [40].

Consistent with our results, some studies show that [28, 41] marriage is associated with nicotine dependence and tobacco consumption. Women above 30 years of age, being married, and living in a joint family were more likely to consume tobacco [42]. One study among heroin and cocaine users also suggests that regular and heavy cigarette smokers were more likely to have a history of a prior marriage [43]. Several factors, including smoking behaviors of the family members (especially their spouses) of the MA users and marital conflict, may serve as interpretation of potential influence of marriage on nicotine dependence, yet more studies are necessary to offer more information. Several studies suggest that spouse plays an important role in one's change of smoking behaviors [44, 45]. One study found that marital conflict was related to nicotine dependence among women and marital satisfaction was also found to be different between nicotine-dependent and nondependent women participants in the US naval services [41].

### 4.3. Relationship of Nicotine Dependence and MA-Related Subjective Effects (Euphoria and Sexual Impulse)

Although the difference in some subgroups according to gender and dose of MA was not significant, higher nicotine dependence was generally found to be associated with self-reported stronger euphoria and sexual impulse in our study, which suggested that nicotine might enhance the subjective and physiological effects of MA. Similar findings were detected among cocaine users in several previous studies. In a study [32] on cocaine users, participants were asked “Does nicotine affect the HIGH that you experience from cocaine?” and “Does nicotine affect your desire for cocaine?” (−5, reduces effect; 0, no change; +5, increases effect), and the average scores were 1.3 ± 0.2 and 0.8 ± 0.2, respectively. This suggested that nicotine would produce a small increase in the high experienced when using cocaine and a small increase in the desire to use cocaine [32].

Methamphetamine (MA), like other stimulants, primarily produces its reinforcing and psychostimulant actions through the brain reward circuit, whose main components are thought to include the mesocorticolimbic dopamine system [10]. Dopamine release mediates the rewarding effects of stimulants, either facilitating release (e.g., amphetamines) or blocking uptake (e.g., cocaine) of dopamine [10, 46, 47]. MA produces its acute effects of modulating dopamine release by acting at the vesicular monoamine transporter-2 and the plasmalemmal dopamine transporter, and acute exposure to MA typically results in large increases in dopamine levels in the dorsal striatum [2]. Meanwhile, nicotine seems to produce its reinforcing and psychostimulant effects by enhancing dopaminergic activity through blockade of dopamine reuptake and increasing synaptic dopamine release [48].

One animal experiment found that 5-time nicotine administrations at 3-day intervals developed a significant locomotor stimulant effect and caused an enhanced sensitivity (cross-sensitization) to MA, although nicotine had no effect at first administration [49]. Another animal studyusing guinea pig brain slices shows that nicotine acts directly in striatum where it enhances dopamine release during phasic but not tonic activity, which may serve as a mechanism for nicotine facilitation of reward-related dopamine signals [50]. 

### 4.4. Limitations

Due to the cross-sectional study design, our research cannot confirm that nicotine directly reinforces the subjective effects of MA and MA does increase the dependence on nicotine. Moreover, detailed information on smoking, such as the reason for smoking cigarettes and the initial age of smoking, was not included in our research plan. Thirdly, reporting bias and recall bias were possible in our study, which may result in inaccurate assessment of the MA use pattern. Finally, our study conducted in Beijing and Guangdong can provide some useful clues of characteristics of Chinese MA users, but we did not obtain information on household registration in our study so we did not discuss the difference between the two provincial regions. Studies covering bigger geographical areas with larger sample size would provide more information on cigarette smoking behaviors and nicotine dependence among MA users and difference between different areas can be further analyzed as well.

## 5. Conclusions

Our findings suggest that difference in social-demographical characteristics (gender and marriage) and MA use pattern (MA dosage and ever-use of other psychoactive substances) may result in disparities in nicotine dependence among MA users. Understanding of the status of nicotine dependence would help us better comprehend MA-related subjective feelings as MA users with severe nicotine dependence might experience stronger euphoria and sexual impulse after MA administration. Prospective studies and animal experiments are required to provide more confirmation of potential risk factors for nicotine dependence and to explore its association with MA-related subjective effects. Our findings suggest that we should consider gender, marital status, and use of other psychoactive substances when providing health education on tobacco control and smoking cessation among MA users, which may be beneficial to improve individualized health promotion.

## Figures and Tables

**Table 1:** Scoring system of Fagerström Test for Nicotine Dependence.

Item^a^	Answers	Points	Response	
			Number	%
(1) How soon after you woke up did you smoke your first cigarette?	Within 5 min	3	127	35.7
	6–30 min	2	140	39.3
	31–60 min	1	41	11.5
	After 60 min	0	48	13.5
(2) Did you find it difficult to refrain from smoking in places where it is forbidden?	Yes	1	154	43.3
	No	0	202	56.7
(3) Which cigarette would you most hate to give up?	The first one in the morning	1	188	52.8
	Any other	0	168	47.2
(4) How many cigarettes per day did you smoke?	10 or less	0	73	20.5
	11–20	1	163	45.8
	21–30	2	56	15.7
	31 or more	3	64	18.0
(5) Did you smoke cigarette more frequently during the first hours after awakening than during the rest of the day?	Yes	1	120	33.7
	No	0	236	66.3
(6) Did you smoke cigarettes even if you were ill in bed much of the day?	Yes	1	207	58.1
	No	0	149	41.9

Total scores	Low or medium (LMD)	0–2	59	16.6
		3-4	91	25.6
		5	47	13.2
	Severe or high (HD)	6-7	78	21.9
		8–10	81	22.8

^a^All the 6 questions were answered according to their cigarette smoking behaviors after participants began using MA usually.

**Table 2:** Association between social-demographic characteristics and nicotine dependence among MA users.

	FTND					
	LMD (≤5)		HD (>5)		*χ* ^2^	P
	n	%	*n*	%		
Gender					10.838	0.001
Male	137	50.6	134	49.4		
Female	59	71.1	24	28.9		
Education					0.076	0.995
Primary school or illiterate	28	56.0	22	44.0		
Junior high school	98	55.4	79	44.6		
Senior high school	48	54.5	40	45.5		
College or above	20	57.1	15	42.9		
Ethnicity					0.017	0.895
Han	169	53.5	147	46.5		
Other	11	55.0	9	45.0		
Marriage status^a^					11.334	0.001
Unmarried	145	61.7	90	38.3		
Married	52	43.0	69	57.0		
Occupation					4.489	0.106
Unemployed	90	62.1	55	37.9		
Private businessmen	57	50.9	55	49.1		
Other	50	50.5	49	49.5		
Age (years)	30.7 ± 7.5		31.9 ± 7.6		−1.560	0.120
Age of initial use (years)	27.8 ± 7.6		28.9 ± 7.9		−1.403	0.162

^a^Unmarried refers to being single or ever married but divorced or separated.

**Table 3:** Association between drug-use pattern and nicotine dependence among MA users.

	FTND					
	LMD (≤5)		HD (>5)		*χ* ^2^	*P*
	*n*	%	*n*	%		
Treatment times					0.725	0.395
First entry	130	57.0	98	43.0		
More than once	67	52.3	61	47.7		
Cumulative use time					0.410	0.522
≤8 months	102	56.7	78	43.3		
>8 months	90	53.3	79	46.7		
Administration route of MA						
Smoking	193	55.1	157	44.9		0.696
Intravenous injection	2	100.0	0	0		0.504
Usual dose of MA					10.776	0.001
≤0.2 g	117	63.2	68	36.8		
>0.2 g	76	45.8	90	54.2		
Ever-use of heroin^a^					7.582	0.006
No	156	59.1	108	40.9		
Yes	28	40.6	41	59.4		
Ever-use of MDMA (‘‘Ecstasy")^a^					8.178	0.004
No	169	58.9	118	41.1		
Yes	27	39.7	41	60.3		
Ever-use of marijuana^a^					2.396	0.122
No	181	56.60	139	43.40		
Yes	15	42.90	20	57.10		
Ever-use of ketamine^a^					4.290	0.038
No	162	58.1	117	41.9		
Yes	34	44.7	42	55.3		
Ever-use of alcohol^a^					6.729	0.009
No	102	63.4	59	36.6		
Yes	91	49.5	93	50.5		

^a^Ever-use means the participant had ever used the substance before the survey started, but not necessarily concurrent with MA.

**Table 4:** Binary logistic regression of risk factors for severe nicotine dependence (FTND > 5).

Risk factors	*n*	OR	95% CI of OR
Gender			
Male	271	2.615	1.345–5.086
Female	83	1	
Marriage			
Unmarried^a^	235	1	
Married	121	1.938	1.159–3.239
Ever-use of ketamine^b^			
No	279	1	
Yes	76	2.044	1.074–3.890
Ever-use of alcohol^b^			
Yes	184	1.699	1.057–2.732
No	161	1	
Usual dose of MA			
≤0.2 g	185	1	
>0.2 g	166	1.659	1.015–2.712

^a^Unmarried refers to being single or ever married but divorced or separated.

^b^Ever-use means the participant had ever used the substance but not necessarily concurrent with MA before the survey started.

**Table 5:** MA-related subjective effects (VAS score) between two nicotine-dependent groups.

Subjective feelings	Subgroup factors	FTND		*Z*	P
		LMD (≤5)	HD (>5)		
Euphoria of onset use		3.00	6.70	−7.613	<0.001
Gender	Male	3.50	7.10	−6.214	<0.001
	Female	1.40	4.10	−2.453	0.014
Dose of MA	≤0.2 g	2.50	6.00	−5.127	<0.001
	>0.2 g	4.65	7.10	−3.814	<0.001
Euphoria of usual use		3.65	5.20	−5.000	<0.001
Gender	Male	4.50	5.20	−3.062	0.002
	Female	2.10	4.70	−4.004	<0.001
Dose of MA	≤0.2 g	2.70	5.15	−3.796	<0.001
	>0.2 g	4.70	5.50	−1.956	0.050
Sexual impulse after using MA		5.90	7.60	−5.870	<0.001
Gender	Male	6.30	7.70	−5.655	<0.001
	Female	3.50	5.35	−1.783	0.075
Dose of MA	≤0.2 g	5.30	8.40	−5.689	<0.001
	>0.2 g	6.45	7.15	−2.186	0.029
